# Regressing calcifications in the carotid artery: What’s going on? A case series

**DOI:** 10.1016/j.radcr.2025.05.108

**Published:** 2025-07-04

**Authors:** Dianne H.K. van Dam-Nolen, Taihra Zadi, M. Eline Kooi, Jeroen Hendrikse, Paul J. Nederkoorn, Aad van der Lugt, Daniel Bos

**Affiliations:** a Department of Radiology and Nuclear Medicine, Erasmus University Medical Center Rotterdam, Rotterdam, The Netherlands; b Department of Radiology and Nuclear Medicine, CARIM School for Cardiovascular Diseases, Maastricht University Medical Center, Maastricht, The Netherlands; c Department of Radiology, University Medical Center Utrecht, Utrecht, The Netherlands; d Department of Neurology, Amsterdam UMC, University of Amsterdam, Amsterdam Cardiovascular Science, Amsterdam, The Netherlands; e Department of Epidemiology, Erasmus University Medical Center Rotterdam, Rotterdam, The Netherlands

**Keywords:** Atherosclerosis, Calcifications, Carotid artery, Computed tomography angiography, Ischemic stroke, Serial imaging

## Abstract

This case series describes a finding that carotid calcifications could decrease during follow-up, as the predominant idea is that calcifications either progress or stabilize over time. This novel finding is of great interest considering that potential regression of calcifications could affect prevention strategies for atherosclerosis and ischemic stroke. We describe 3 patients (3 men, age ranging from 72 to 75 years) with decrease in carotid calcifications during 2-year follow-up. All 3 patients had macrocalcifications and used different medications during follow-up. This remarkable finding gives new insights in the natural course of atherosclerosis as a main cause of ischemic stroke. Further study is needed to investigate whether this is a harmful or beneficial process and to unravel pathophysiological mechanisms.

## Background

Carotid atherosclerosis is a major cause of ischemic stroke, and thereby contributes to considerable morbidity and mortality [1,2]. Atherosclerotic carotid plaques vary greatly in size, composition, and structure, but the presence of calcifications is very common in more advanced plaques [3,4]. Over the years, the presence and amount of calcifications has often served as a marker of atherosclerotic disease and as a risk indicator of stroke events, but its relation with cardiovascular risk remains complex [5,6]. The predominant hypothesis regarding calcifications is that these generally either progress or stabilize over time, yet, evidence for this hypothesis from serial imaging studies remains scarce. Recently, we found that carotid calcifications could also decrease during follow-up. The aim of this case series is to explore and discuss this novel finding, in the belief that this could be of great interest considering that potential regression of calcifications could affect prevention strategies.

## Case presentation

**Fig. 1:**
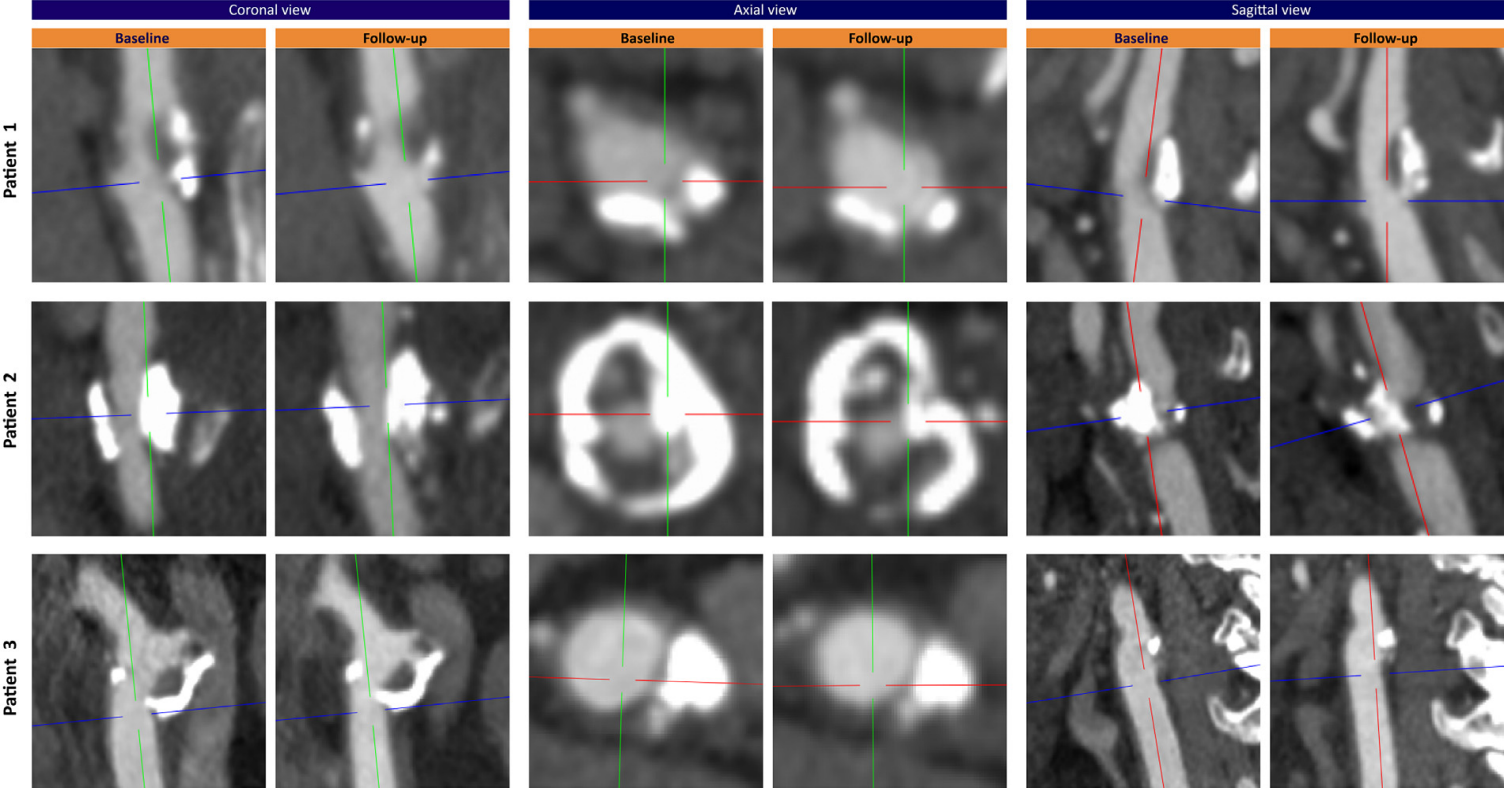
Baseline and follow-up CTA of patients with decreasing ECAC. CTA images in axial, sagittal, and coronal views at baseline and 2-year follow-up of patients with decrease in ECAC.

An overview of patients’ clinical characteristics at baseline and medication use are shown in Table 1. Table 2 shows the plaque characteristics at baseline. In Figure 1 CTA images in axial, sagittal, and coronal views are compared for baseline and follow-up. We performed state-of-the-art carotid plaque imaging at baseline and after 2 years follow-up using contrast-enhanced CTA and MRI [7]. We assessed extracranial carotid artery calcification (ECAC) volumes within 3 cm proximal and distal to the carotid bifurcation with a custom-made plug-in for the software ImageJ (National Institutes of Health, Bethesda, Maryland). A threshold of 600 Hounsfield units was used to differentiate calcifications from contrast material.

**Table 1:** Clinical characteristics of patients with decrease in ECAC during 2-year follow-up.

Variable	Patient 1	Patient 2	Patient 3
Clinical characteristics at baseline			
Age, years	72	75	72
Sex	Male	Male	Male
Ethnicity	Caucasian	Caucasian	Caucasian
Diabetes mellitus	No	No	No
Hypertension	No	Yes	Yes
Hypercholesterolemia	No	Yes	Yes
BMI, kg/m^2^	24.1	24.3	36.2
Smoker	Former	Former	Former
Alcohol abuse	No	No	No
History of CVD	Yes^a^	No	Yes^b^
History of malignancy	No	No	Yes^c^
Laboratory values at baseline			
LDL (mg/dL)	96.7	100.5	189.5
HDL (mg/dL)	61.9	44.1	38.7
Triglycerides (mg/dL)	125.8	88.6	372.0
Total cholesterol (mg/dL)	185.6	162.4	274.6
Lipoprotein(a) (mg/dL)	Unknown	59.8	3.0
Medication use at discharge^d^			
- Alimentary tract and metabolism		Omeprazole	Hydrocortisone Pantoprazole
- Blood and blood-forming organs	Clopidogrel	Carbasalate calcium Dipyridamole	Carbasalate calcium Dipyridamole
- Cardiovascular system	Simvastatin	Lisinopril Simvastatin	Metoprolol Simvastatin
- Genito-urinary system and sex hormones	Tamsulosin		Testosterone
- Hormonal preparations, excl. sex hormones and insulins			Levothyroxine
- Anti-infectives for systemic use		Cotrimoxazole	
- Nervous system		Primidone	

a Previous amaurosis fugax.

b Ischemic heart disease and peripheral artery disease.

c Pituitary metastasis of unknown primary tumor which was treated with stereotactic radiotherapy.

d At discharge when treated for their baseline event. BMI, body mass index; CVD, cardiovascular disease; LDL, low-density lipoprotein; HDL, high-density lipoprotein.

**Table 2:** Plaque characteristics at baseline of patients with decrease in ECAC during 2-year follow-up.

Variable	Patient 1	Patient 2	Patient 3
Side of plaque	Right	Right	Left
Plaque ipsilateral to index event	Yes	Yes	No
Plaque size			
Degree of stenosis (ECST, %)^a^	65	81	19
Degree of stenosis (NASCET, %)^a^	20	40	0
Total plaque volume (mm^3^)^b^	1238	2086	870
Plaque components			
Presence of calcifications^a^	Yes	Yes	Yes
Percentage of calcifications^a^	14.7%	14.4%	17.8%
Presence of lipid-rich necrotic core^b^	Yes	Yes	No
Percentage of lipid-rich necrotic core^b^	12.7%	3.9%	n.a.
Presence of intraplaque hemorrhage^b^	Yes	No	No
Percentage of intraplaque hemorrhage^b^	7.9%	n.a.	n.a.
Plaque morphology			
Plaque ulceration^a^	No	No	No
Thin-or-ruptured fibrous cap^b^	Yes	No	No

a Degree of stenosis, percentage and presence of calcifications, and plaque ulceration are assessed with CTA.

b Total plaque volume presence and percentage of lipid-rich necrotic core and intraplaque hemorrhage, and thin-or-ruptured fibrous cap are assessed with MRI. ECST, European Carotid Surgery Trial; NASCET, North American Symptomatic Carotid Endarterectomy Trial.

### Patient 1

The first patient is a 72-year-old, formerly smoking man with a medical history of amaurosis fugax. He was admitted to the hospital with an ischemic stroke in the right hemisphere. Carotid MRI at baseline showed an ipsilateral plaque with calcifications, a lipid-rich necrotic core (LRNC) including intraplaque hemorrhage (IPH), and a thin-or-ruptured fibrous cap (TRFC), causing a stenosis of 65% (according to the European Carotid Surgery Trial criteria). We found that the calcification decreased during follow-up (baseline ECAC volume: 64.7 mm^3^; follow-up ECAC volume: 35.3 mm^3^), predominantly in the proximal region of the plaque. The distal part of the plaque conversely showed larger calcifications at the follow-up scan.

### Patient 2

The second patient concerns a 75-year-old, formerly smoking, male patient with hypertension and hypercholesterolemia. He was referred to the out-patient clinic due to a right-sided amaurosis fugax. At baseline he had an ipsilateral carotid plaque with calcifications and a small LRNC, without IPH, causing a stenosis of 81%. During follow-up the calcification decreased, especially in the bulk of the plaque (baseline ECAC volume: 288.6 mm^3^; follow-up ECAC volume: 127.1 mm^3^). However, new IPH and an increased LRNC (from 3.9 to 10.8%; 80.8 to 154.6 mm^3^) was found at the follow-up scan.

### Patient 3

The third patient is a 72-year-old, formerly smoking, male patient, also with hypertension and hypercholesterolemia. He was admitted to the hospital with a TIA in the right hemisphere. During follow-up he had decrease in calcifications in the contralateral carotid plaque (baseline ECAC volume: 364.9 mm^3^; follow-up ECAC volume: 203.8 mm^3^). At baseline, this plaque was composed of only calcifications and fibrous tissue, causing a stenosis of 19%. The decrease in calcification was seen in all regions of the plaque (Fig. 1 shows decrease in the proximal region). It seemed that in the ipsilateral plaque also some decrease in calcifications occurred during follow-up, but it was not that evident as on the contralateral side.

## Discussion and conclusions

We found that carotid calcifications can regress over time. This is a new and remarkable finding. Although previous studies have described carotid plaque regression (often to evaluate statin therapies) [8,9], no studies have explicitly reported about carotid calcification regression. In the coronary field, studies particularly focusing on statin use have shown that coronary calcifications could both decrease and increase [10,11]. However, it is important to realize that the development of calcifications may differ across different vessel beds within 1 individual, as evidenced by merely weak-to-moderate correlations [12,13].

Increase in calcifications have possible stabilizing effects [10]. It is believed that especially macrocalcifications rather than micro or spotty calcifications contribute to these potential protective effects [14]. All 3 patients in our study had macrocalcifications which makes the finding of a decrease in calcifications more intriguing.

The process of calcifications is enigmatic. Multiple factors like lipoproteins and inflammatory cells play a role and medications could have pleiotropic effects. There are some notable clues in our case series. First, the third patient used long-term hydrocortisone during the whole follow-up because of a pituitary tumor. We hypothesize that the use of anti-inflammatory medication could be a factor mitigating carotid calcifications. Based on previous literature, we already know that anti-inflammatory medication might delay atherosclerosis progression [15], yet it may also cause decrease in calcifications. The fact that decrease was seen in the whole plaque and possibly on both sides, strengthens this idea. Secondly, 2 patients (no. 1 and 2) had low LDL levels while using statins. Raggi et al. reported in a study among patients without manifest coronary artery disease that only in treated patients who achieved an LDL level <120 mg/dL calcification regression was seen [16]. The use of statins and relatively low LDL levels in these 2 patients may have contributed to the calcification decrease. However, it is remarkable that this happened in probable late-stage macrocalcification. Besides this, the decrease in calcifications happened in 2 patients (no. 1 and 2) at specific regions of the calcification, suggesting a more local process or suggesting that the calcification at those points was more sensitive for changing due to an earlier stage. It is obvious that our findings provide more questions than answers.

Besides the question about the pathophysiology, these findings also raise the question whether regressing calcifications are beneficial or harmful. When total plaque size is regressing it could be a beneficial process reducing the risk of stroke. However, when calcifications are decreasing but components like IPH and LRNC are increasing, the plaque could become more vulnerable and prone to rupture. In light of preventing stroke and new therapies, it is important to investigate these questions further and more deeply in future research and in larger cohorts.

In conclusion, in this case series, we showed 3 examples of decreasing carotid calcifications during follow-up which gives new insights in the natural course of atherosclerosis. This remarkable finding needs to be validated in other and larger multicenter cohorts and studied further to investigate whether this is a harmful or beneficial process and to unravel underlying pathophysiological mechanisms.

## Ethics approval and consent for participate

Institutional Review Board approval was obtained (Medisch Ethische Commissie azM/UM, Maastricht, the Netherlands, approval number NL29116.068.09/MEC 09-2-082) and all patients gave written informed consent for participation in this study and for publication of collected information regarding their medical case and imaging. The study was performed in accordance with the principles of the Declaration of Helsinki.

## Availability of data and materials

The data underlying this article cannot be shared publicly due to the privacy of individuals that participated in this study. The data will be shared on reasonable request to the corresponding author.

## Author contributions

DvDN: Conceptualization, Formal analysis, Methodology, Writing—original draft, Visualization. TZ: Conceptualization, Formal analysis, Visualization. MEK: Funding acquisition, Writing—review and editing. JH: Funding acquisition, Writing—review and editing. PN: Funding acquisition, Writing—review and editing. AvdL: Funding acquisition, Supervision, Writing—review and editing. DB: Methodology, Supervision, Writing—review and editing.

## Patient consent

Included patients gave informed consent for publication of collected information regarding their medical case and imaging in context of the PARISK study.
